# Web-of-Objects (WoO)-Based Context Aware Emergency Fire Management Systems for the Internet of Things

**DOI:** 10.3390/s140202944

**Published:** 2014-02-13

**Authors:** Zia Ush Shamszaman, Safina Showkat Ara, Ilyoung Chong, Youn Kwae Jeong

**Affiliations:** 1 Department of CICE, Hankuk University of Foreign Studies, Yongin-si 449-791, Korea; E-Mails: shamyo@ymail.com (Z.U.S.); safina_ara@yahoo.com (S.S.A.); 2 Electronics and Telecommunication Research Institute (ETRI), Daejon 305-700, Korea; E-Mail: ykjeong@etri.re.kr

**Keywords:** Internet of Things (IoT), Web of Things (WoT), emergency fire management, context awareness, ViOs, semantic ontology

## Abstract

Recent advancements in the Internet of Things (IoT) and the Web of Things (WoT) accompany a smart life where real world objects, including sensing devices, are interconnected with each other. The Web representation of smart objects empowers innovative applications and services for various domains. To accelerate this approach, Web of Objects (WoO) focuses on the implementation aspects of bringing the assorted real world objects to the Web applications. In this paper; we propose an emergency fire management system in the WoO infrastructure. Consequently, we integrate the formation and management of Virtual Objects (ViO) which are derived from real world physical objects and are virtually connected with each other into the semantic ontology model. The charm of using the semantic ontology is that it allows information reusability, extensibility and interoperability, which enable ViOs to uphold orchestration, federation, collaboration and harmonization. Our system is context aware, as it receives contextual environmental information from distributed sensors and detects emergency situations. To handle a fire emergency, we present a decision support tool for the emergency fire management team. The previous fire incident log is the basis of the decision support system. A log repository collects all the emergency fire incident logs from ViOs and stores them in a repository.

## 1. Introduction

There is a robust trend en route toward a world of sensors with everyday objects equipped with embedded data and communications capabilities in the Internet of Things. This will create a range of potential new services in many different domains such as smart homes, e-health, automotive transportation and logistics, environmental monitoring, emergency management services, *etc.* [1–4]. Research in this area has gained momentum and is backed by the collaborative efforts of academia, industry, and standardization bodies in various communities, although current devices and communication infrastructures characterized by proprietary protocols but a lack of common standards both on the network and application level prevent the realization of this vision.

Internet of Things (IoT) realizes the concept of pervasive and ubiquitous computing with the inclusion of sensors, actuators, mobile devices and even product information tags using RFID. Within the scope of IoT, all these “smart things” are addressable to interact with their environment, and react to any event with other things/objects to accomplish assigned tasks [1–4]. The basic motto of IoT is connecting with anything by anyone at any time from any place. Sensors and actuators are typical examples of such smart things. IoT itself is a consolidation of various technologies as depicted in [1].

Reference [5] proposes new approach, Web of Objects (WoO), which provides the feature of combining the characteristics of web applications and the various virtual objects mapped from multiple things. Further, WoO supports the features to collaborate not only things but humans, services, resources, various types of tangible things as virtual objects through the use of semantic ontology to promote the composition of objects [6].

The goal of the WoO is to deliver a service infrastructure that simplifies the management of the smart service environments able to provide a service that integrates various technologies like cloud computing and social networking. Hence, WoO allows the reuse of existing web technologies to build new applications and services by attaching smart objects to the Web. In this way, smart objects are abstracted as web services and seamlessly integrated into the living world of the Web, while services are discovered, composed and executed as needed.

**Figure 1.**
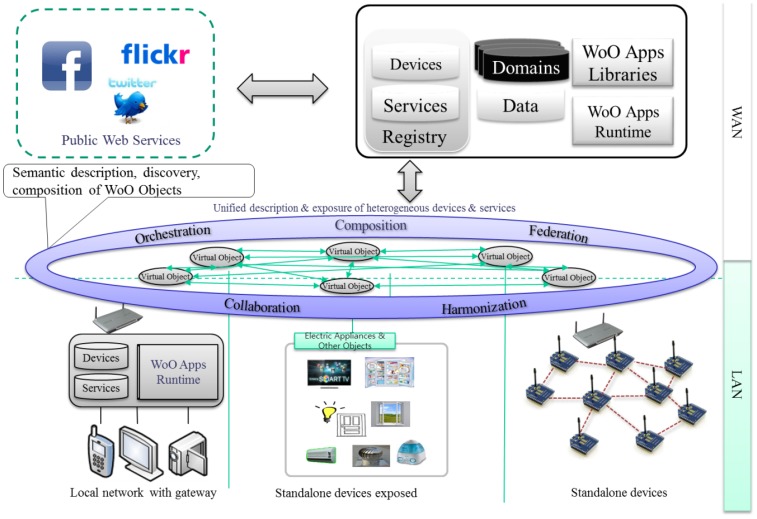
Context of Web of Objects (WoO). Physical objects in real world are represented as virtual objects, and virtual objects are utilized in collaboration, harmonization and composition with each other to form new application features to satisfy the service requirements initiated by users and operators.

Subsequently, in Figure 1, Web of Objects (WoO) [6–8] facilitates diverse disseminated applications to promote smart realms which are presently requested for more intelligent operation of distributed applications. The integrated design based on Web service environments of WoO supports an efficient migration to an identical resource-efficient infrastructure, uniform data and a comprehensive semantic description in service operations, administration and management processes.

In Figure 1 we present the WoO concept and its context to identify the functionalities in local and backend level. Local level concerns the connections of objects at the LAN level. Challenges at this level are object connection, capabilities discovery, logic execution, exposure of features in shared formalisms and technologies.

On the other hand, backend level concerns the infrastructure backend that provide WAN connectivity. The WoO backend provides features by allowing the design, execution and support of IoT based applications. The key aspects are discovery of objects at the WAN level, allowing access to the functional and non-functional capabilities of connected objects, logic execution (centralized or decentralized), and semantic adaptation of objects and exchanged data for cross-domain interaction, interaction of WoO with external services.

In this paper, we do not consider the WAN as part of WoO. Instead, we consider that objects are connected at the local level through a smart gateway which is linked to the application server in the backend to provide the needed service. Hence, in this paper, we intend to present an emergency fire management system by using WoO Virtual Objects (ViOs), as well as the incident log from those ViOs in order to accelerate and enrich the scope of WoO. WoO portrays every entity as an object, for example, temperature (temperature sensor), gas (gas sensor), devices, human/user, time, date, etc. Hence, to satisfy the requirements of WoO, we present physical object representations in a virtual world named ViOs. All the ViOs are semantically related to each other with the semantic description. A semantic ontology model for emergency fire management is presented in this paper as well. Hereafter, each ViO is the abstraction of a particular physical object. Likewise, more than one ViO can form new ViOs according to the service requirements. As new ViOs are created from the original ViOs, they inherit the resources, features and characteristics of the original ViOs.

Fire symptom detection is performed in the application server of WoO by measuring the changes in the atmosphere such as the temperature level, smoke level and gas level. The distributed pool of sensors senses the environment and transfers collected values immediately to the Application Server (AS) through the WoO gateway. After analyzing those real world environment data according to a predefined policy, AS detects an emergency fire situation. After the detection of a fire incident, the fire management team takes actions based on previously occurred similar types of fire incidents. These previous conditions data are retrieved from the log repository [9]. The emergency situation log is collected from ViOs and is stored in the log repository. We characterize a shopping mall fire incident to portray an emergency fire management system, but it is not limited to any specific type of organization or territory.

If simplicity and intuitiveness can be obtained for the end users—not only in using various applications while interacting with their physical surroundings, but also in creating new experiences via the same tangible real-world interactivity—then indeed, a potentially large market exists for actors in the service-enabling space, ranging from domain-specific application service providers down to Internet-of-things infrastructure interconnectivity providers and network operators. We expect that the dynamics of the long tail marketplace for applications (and application components) will result in the most relevant components become part of the enabling substrate. As such, today's applications will evolve into tomorrow's service infrastructure.

## 2. Related Work

Several attempts have been explored for incorporating applications and services into the real world. Service Oriented Device & Delivery Architecture (SODA) [10,11] is an adaptation of a service-oriented architecture (SOA). The SODA approach for designing and building distributed software is to adapt a wide range of physical devices into disseminated IT inventiveness systems.

SOA is a well-known IoT middleware solution approach. However, a widely recognized layered architecture is ignored in SOA and faces problems of abstracting objects' functionalities and communications capabilities which are required for service composition. ITEA2 OSAMI commons [12,13] project have shown the basic design of SOA-oriented platforms. This knowledge can be applicable in the context of the global roadmap of the WoO.

DiYSE [14,15] is another approach that is easy to use for normal users, but rarely suitable for professionals. Moreover, its object representation and business process model cannot handle complexity due to the lack of dynamicity in connecting objects as well as creating new services. Hence, WoO is intended to enhance that expertise to the professional level as well as to handle complex workflows for creating new services. It ensures interoperability by providing a smart gateway in the WoO architecture and creating this ViOs according to the application requirements.

Internet of Things Architecture (IoT-A), an ongoing project, reference [16] proposes an architectural reference model together with the definition of an initial set of key building blocks. Advancements in miniaturization, energy harvesting, and the integration of computing and communication components into non-standard substrates enable the implementation of the most science-fiction visions that we have today, but the absence of a uniform and comprehensible architecture could threaten the deployment of the Internet of Things (IoT).

Therefore, the first step towards realizing this vision is the development of an open architecture reference model, providing guidance for the technical decisions. Service management of semantic and context aware semantic service architecture is considered in SemBySem [17] and SemEUse [18], respectively. A dynamic monitoring system, using composite probes, is also connected to the orchestration process, so that QoS-aware late binding can be implemented [19].

The consequent research challenge exploded with the evolution of the IoT domain that has grabbed our attention in the physical object representation in the virtual world. An approach for virtual sensors has been described in [20], but without exposing any implementation details for the user. A virtualization has been focused on in [20] through managing those ViOs to comply with assorted and pervasive environments to ensure context awareness, as well as highly reliable service provision.

A few more research works are concentrated on several technical features as well as on application domains. For example, the SOFIA project [21] tries to produce a semantic interoperability platform to make physical world data available for smart services by establishing links between the physical world and the virtual world. The authors of [22] inspected the process to include ontologies, runtime task models and Belief Desire Intention (BDI) to enable semantic interaction for pervasive computing. On the other hand, the researchers in [23] have considered overt semantics to design illustrated models of association among the devices in a smart home environment. Three diverse levels of abstraction such as resources, entities and resource users with an architectural model have been presented as the main concept of the SENSEI project [24]. Resources are the virtual form of devices, entities are the virtual representation of people, places and things and resource users are the end user who communicates with the resources and entities [25].

The aforementioned examples illustrate that current devices and communication infrastructures characterized by proprietary protocols and a lack of common standards, both at the network and application level, prevent the vision of sharing data among intelligent objects or cooperating in groups to achieve complex goals. We are targeting a Web of Objects (WoO) facilitating smart distributed applications that combine information from different domains currently isolated from each other. In order to facilitate simple development, deployment and operation of smart distributed applications, an integrated design based on a uniform resource-efficient infrastructure, uniform data and service models is stressed and a comprehensive semantic description WoO facilitates easy creation of cross-domain applications able to target goals that have not been envisaged at system deployment time.

In summary, with several projects, standards, prototypes, products and approaches from the industry and academia, it has become possible to publish the real world to the digital world as well as to the Web. We intend to propose a smart environment using the existing approaches and show a scenario to handle an emergency fire management situation. According to our knowledge, even though several systems have been proposed in this way or another, there are still no ViO's log-based fire emergency management systems in the IoT domain.

The rest of this paper is structured as follows: Section 3 introduces the reader to the WoO architecture for emergency fire management. Section 4 presents the object virtualization and management model in WoO. Section 5 describes the context aware emergency management system operation and its related components. A prototype implementation is shown in Section 6, and finally, we conclude this paper in Section 7.

## 3. WoO Architecture for Emergency Fire Management

**Figure 2.**
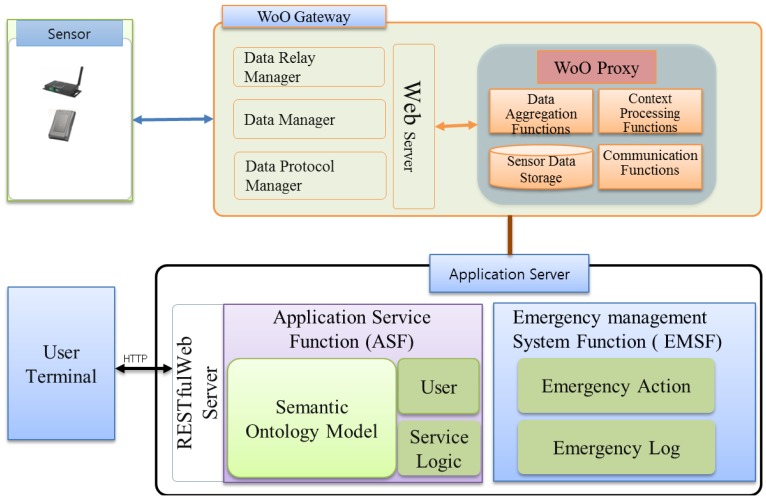
Web of Objects (WoO) architecture for an emergency fire management system. Users can only connect to the Application Server through the App to get a required service.

This paper presents our proposed WoO architecture for fire emergency management systems and subsequently integrates the object virtualization and management approach into the WoO. To ensure the relationships among objects, the semantic ontology model is also presented in the context of WoO. Consequently, the fire-incident log collection procedure is exhibited to tackle the new fire incident. Finally, the emergency fire management system is described, including a prototype implementation. We consider the Web's easy application capability and the IoT's abundance of smart objects. The proposed WoO architecture in this paper uses the web-based service platform structure inspired by the current existing approaches like SemBySem and SemEUSE for the semantic description of objects and services. On the other hand, the overall system architecture is inspired by OSAMI and DiYSE and heterogeneous device management is inspired by IoT-A. This section presents the WoO architecture and semantic ontology model by reusing existing web architectures as platform, smart objects services on the web. Two important issues to achieve by this are: how to integrate physical things into the web and how to make these physical things offer web services. Figure 2 depicts all the components of the WoO architecture. Sensor devices are responsible for collecting data from the real world and sending it to the WoO gateway (the communication medium can be Zigbee, Wi-Fi or Bluetooth). The gateway, on the other hand, maintains a device profile for each and every sensor node and receives raw data from the sensors. Any faulty device profile can be discarded by the WoO gateway and new device profiles can also be added according to the requirements. There is a person with proper authority responsible for configuring the gateway. This part is very sensitive as any missed configuration or failure to address the faulty sensor could result in a failure to understand an emergency situation properly.

The WoO gateway is capable of communicating with sensor nodes through various communication mechanisms and protocols. It also has the capability to relay environmental data to the WoO proxy in active and passive mode. In active mode sensors shall push data to the gateway, and in passive mode the WoO gateway pulla data from sensor devices. The WoO proxy then aggregates this data and transfers it to the AS. Hence, in this thesis, the WoO proxy communicates directly with the AS. There are several parts in the AS such as the Application Service function (ASF), the Emergency Management System Function (EMSF) and the ontology model. EMSF is the main function to handle the emergency situations instantly after a fire incident is detected by the ASF.

We describe the EMSF architecture in Section 5. It explains that ASF and EMSF have been designed within the Application Server (AS) and are connected to each other for the interaction of data and instructions. We consider emergency situations for a shopping mall as a test case in this paper; however, it is not limited only to the shopping mall scenario as simple or no modifications are required to comply with the needs of different organizations. The terminal or client module is the smart phone app or a browser-based client. Through this module, users can access the WoO service infrastructure and receive notifications and alert messages. Detailed emergency management procedures have been described in Section 5.

In order to handle an emergency situation, it is very important to respond as quickly as possible; however, this response is dependent on the available information about the fire incident. Here, we consider information about the fire location, the source of the fire, the number of persons, and the area on fire. After getting this information, the next step for the emergency management team is to take actions like triggering the fire alarm, initiating hotline calling, monitoring the fire affected area, opening exit doors, providing an escape route to the users, providing special assistance for the handicapped and trapped persons, *etc*.

Each type of information is treated as a single object in WoO and all the ViOs are semantically related to each other. Virtual representation of all the objects can be combined with each other to form a new object based on the application requirement. Object virtualization is described in Section 4.

**Figure 3.**
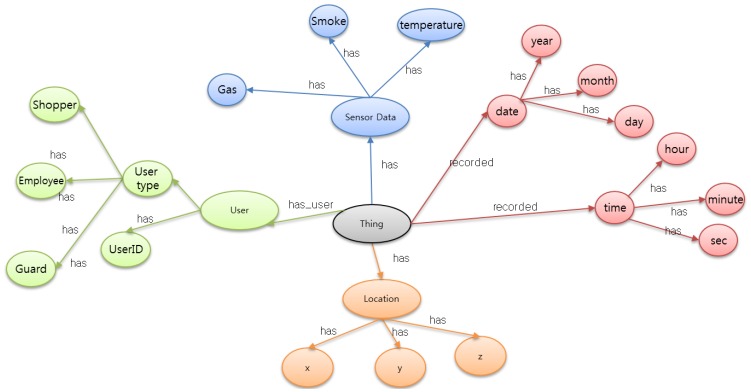
All the required objects to handle a fire emergency situation is considered in this figure. Virtual representation of all objects maintains the semantic relation with each other. For better visualization, all the objects are shown with arc but any kind of combination is possible based on the application requirement.

The emergency fire ontology is based on the concepts of systems, processes, and observations. It supports the description of the physical and processing structure of objects. Objects are not constrained to physical sensing devices only; rather, an object is anything that can provide the value of an occurrence, so a device or computational process or combination could play the role of an object. The representation of an object in the ontology links together what it measures, the physical sensor and its functions and processing. WoO semantic ontology map in Figure 3 shows the relationship of each object to gather data and information according to its specification.

Physical objects are represented as ViOs in WoO. This virtualization concept is based on the WoO semantic ontology model. Ontology modelling [26] has the advantages of information reusability, extensibility, and interoperability which is the key point to maintaining the federation, orchestration, collaboration and harmonization among all the ViOs. These ViOs are capable of collaborating with each other and forming new ViOs according to the service requirements.

In Figure 3, we consider only those objects which are directly related to the emergency fire incident. Sensor devices collect raw data to detect the fire symptom, while the user object has the user type and user-id; furthermore; the user type includes a shopper, an employee and a security guard. When a fire incident happens, all these objects collaborate with each other according to the requirement.

## 4. Object Virtualization in WoO

This section presents the object virtualization and the management to support the virtual matching part in the WoO for emergency fire management. The process consists of three layers of functionalities and each layer comprises different entities that provide the harmonization, federation and orchestration among objects for flexible service to user applications. The object virtualization process ensures the object abstraction and heterogeneity that derives from the vast amount of diverse physical objects, while enhancing flexibility to facilitate the consideration of the views of various users for ensuring proper application provision, business integrity, therefore, maximization of exploiting opportunities.

### 4.1. Layered Approach

**Figure 4.**
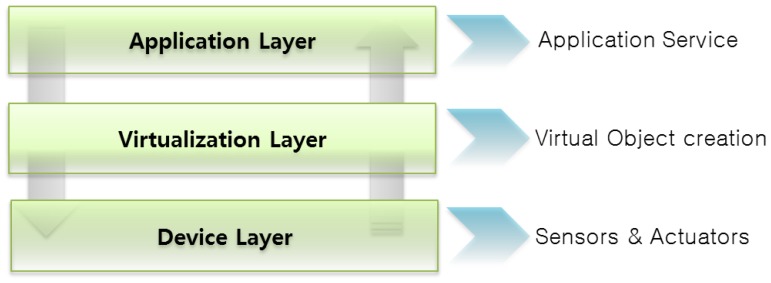
The object virtualization process happens in three layers. After getting the application's requirement from the application layer, the virtualization layer creates ViOs against physical objects. This layer provides the bridging between the physical world and virtual world. On the other hand, the application layer interfaces with the user, and the device layer consists of all the physical objects and gateways of those objects.

Figure 4 shows that the object virtualization concept consists of three layers of functionality. ViOs are reusable for diverse applications and is based on a semantic ontology model. The three proposed defined layers are described in more detail in the following paragraphs.

Device layer is the lowermost layer, consisting of all the sensors, actuators and communication mechanisms. Sensors are connected to the gateway and collect real world raw data like temperature, smoke and gas. Managing these sensor nodes is crucial to the success of the overall system. Wrong readings or failure to sense any environmental values can be very hazardous for the overall system. To address this issue, we consider that an authorized person should be responsible for creating and maintaining a device profile in the WoO gateway.

The virtualization layer defines the creation of ViOs against all the physical objects and provides links between the physical and the virtual world. It comprises virtual representations of sensors and the values that are collected as observations by each ViO. This layer also includes the dynamic creation of ViOs according to the application's requirement as well as the user's requirement. This is actually the combination of two or more ViOs which is formed by following the semantic ontology relation. All the ViOs are harmonized with each other to maintain orchestration. This dynamic federation enables the system to provide on-demand services to users.

The application layer interfaces with the users and also requests the down layer to prepare objects. It comprises functionalities and interfaces for acquiring user requests, preferences and constraints that are taken into account for the automated creation and dynamic configuration of objects, the provision of smart, customized and personalized services. The user can provide a set of requirements for creating a service that may require combining a few virtual objects to satisfy the user's requirement. Furthermore, intelligent mechanisms are subjugated in order to build a dynamic service for users according to their requirements.

### 4.2. Virtual Object Formation and Management

**Figure 5.**
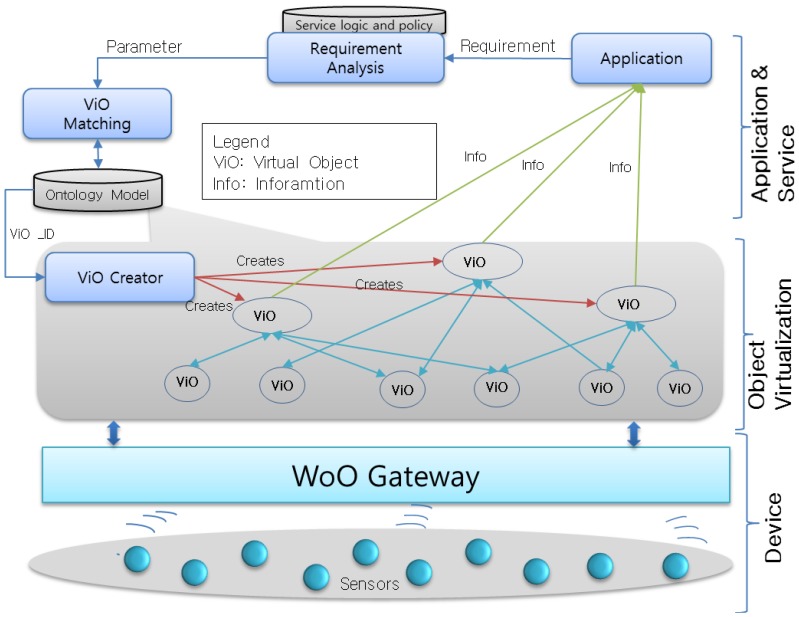
User requirements are analyzed and complied with the system policy. The next step is performed to find and select the required objects to form new ViOs to satisfy the user application requirements. The ontology model defines the relationships among ViOs.

Virtual objects are created and related to each other by a semantic ontology model. This section presents the ViO formation and management method in Figure 5. ViO is the virtual representation of a physical object that contains the information for the description of the physical object, while it is implemented as a program code that is used to link the real world object with the virtual world. Any object, whether it represents the sensors or other physical objects is considered to be a real world physical object and can be described in such a manner that it facilitates in its exploitation and accessibility in the virtual world: for example, a temperature sensor associated with a room and measures the room temperature. On the other hand, shoppers and guards are different types of user objects and can also be represented as ViOs.

A sensor object can also be associated with a user object implicitly and described in the virtual world. For example, if a user needs special assistance during an emergency situation, then the system can associate a guard object (a member of the rescue team during an emergency situation) with the location object to create the assistance service for the user. Essentially, a ViO provides an abstract representation of the features and capabilities of all the objects in the virtual world.

Formation of ViO in our implementation is defined as Resource Description Framework (RDF) [27–29] into a XML file. Prototype implementation is described in Section 6. The ViO matching module matches required objects in the ontology model according to the application's requirements. The ViO creator creates new ViOs with an identity which are actually the combination of existing ViOs for a specific service. Each new ViO will get a unique identity, *i.e.*, ViO-ID.

**Figure 6.**
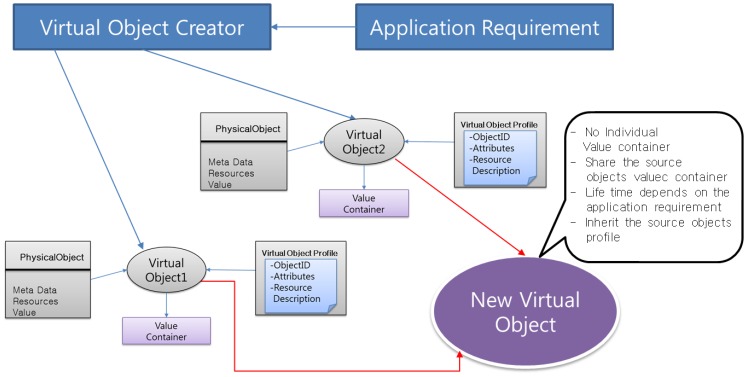
Two ViOs are combined to create a new ViO. Virtual objects have a profile and value container to keep the recent value. But the New ViO does not have any value container as it inherits all the properties of its source ViOs.

Dynamically created new objects are semantically interoperable ViOs that are combined in order to offer services that will fulfill an application request from the user. Figure 6 shows that dynamically created new ViOs inherit the functions and features of the source ViOs. Moreover, it combines them for the provision of composite services by maintaining orchestration with other related objects. These new objects are capable of being reused, even out of the context for which it was initially created. One or many new objects may be used in order to compose an application providing a set of services in accordance with the user requirements.

However, the new ViO does not have any individual value container; rather, it shares the source ViOs' value containers as well as other features. This inheritance allows the ViO to be very flexible and scalable compared to the original ViOs from which the new ViOs are created. For better understanding, we show the steps of new ViO formation in the next paragraph. The steps of new ViO creation are as follows:

Step 1. Receive service requirement from the user application
Step 2. Analyze the requirement and check with the policy
Step 3. Find the required objects and match them with the available objects in the ontology model
Step 4. Create a new ViO by combining existing objects
Step 5. Send the new ViO's data to the application
Step 6. Save the new ViO or destroy it according to the requirement

Lifetime of new ViO depends on the requirement of the application, and new ViOs will be stored or destroyed according the application requirements. As we already have mentioned that all the ViOs are described through semantically enriched information, dynamically created new ViOs are not directly-matched physical objects; rather, they are the combination of a few existing ViOs; hence, they are represented as the combination of ViOs in the program code.

## 5. Context Aware Emergency Management System

Whenever there is a fire emergency, there is neither much time nor much data to sit, analyze and decide on the course of action by the emergency management team. Hence, instead of a complex model, simple context aware methods are considered to determine the course of action during a fire emergency.

**Figure 7.**
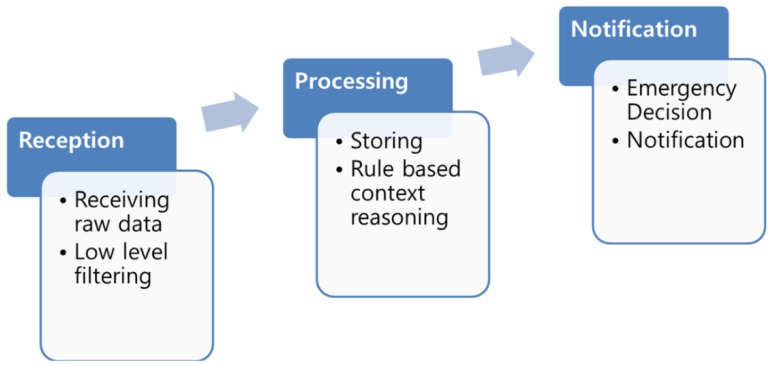
Context awareness is defined as receiving the raw data from the sensor, filter them and then process them based on the rules to detect the fire symptom and then notify the users and other parts of the system like EMSF.

Context Awareness in Figure 7 is an understanding of what is going on around, perception of the elements in the environment within a volume of time and space, the comprehension and the projection of their status [30].

To provide efficient management of emergency situations, it is necessary to introduce autonomy in systems for gathering, processing, managing and provisioning context information, which is termed context awareness in our systems. One of the goals of context awareness is making capability according to the emergency situation detected by the ASF. Context aware emergency management systems allow the dynamic formation of ViOs according to the context information sources when they are needed.

Each sensor can send the sensed value to the ASF and then the ASF processes those data to evaluate the information in order to make decisions about the situations. Here, Context Awareness is satisfied by the sensors and the ASF. However, in the WoO, the user application does not directly connect to the sensor nodes; instead, they receive all the context aware services from the ASF.

Semantic Ontology in WoO provides a relation among objects, which is used to model a situation, *i.e.*, the type of objects that exist, and their properties and relationships. Semantic Ontology in WoO provides a relation among objects, which is used to model a situation, *i.e.*, the type of a reasoner that can check the validity of the modelled domain and draw inferences by applying the rules and axioms for constraints, restrictions, actions which are finally designate the context awareness.

### Operation Method

It has been found that fire incident management personnel acquire experience and knowledge by attending different fire incidents and studying certain procedures and standard incidents. However, since indistinguishable repetition of certain situations is sporadic, responsible persons try to handle a fire situation based on their experience, which is congregated from analogous situations, by equating the similarity of the situations with respect to certain factors representing the situation.

**Figure 8.**
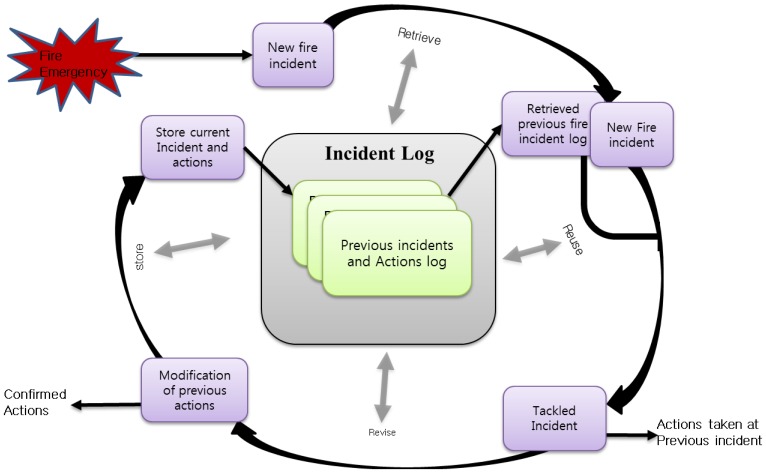
Log-based emergency fire management cycle shows the method to handle the emergency situation and store current incident logs in the log repository.

Figure 8 shows that our proposed method follow the below mentioned steps to handle an emergency fire incident.

- Determine the situation based on the current available data;
- Retrieve a similar incident from the emergency situation log management repository;
- Follow the previous actions or customize the previous actions instantly.

**Figure 9.**
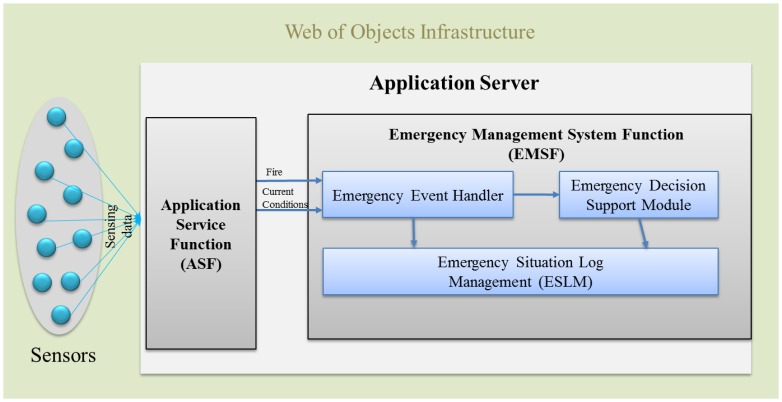
The application server contains the ASF and EMSF. The ASF collects sensed data through the WoO gateway and detects the emergency fire incident. The Emergency Event Handler (EEH) receives notifications from the ASF and informs the Emergency Decision Support Module (EDSM) for further action. The EDSM instructs the ESLM to retrieve similar conditions from the log and store the current incident log to the repository.

Due to the complexity of fire emergencies, the interdependencies among decisions and actions are often neglected despite their importance [31]. EMSF in Figure 9 aims to associate decision support modules to address this issue where time and decisions are interdependent. It is well known that the timing of decisions can influence their consequences. To account for the requirements in different phases of an emergency, two approaches are considered: a collection of real-time fire emergency situation data and settling the management actions based on the previous incident log.

It is worth mentioning here that predefined actions can be injected by concerned personnel for a new emergency management system with no previous fire emergency log. However, at least in this paper, our consideration is not an empty system.

Figure 9 shows that the Emergency Management System Function (EMSF) is used as a decision support tool by providing similar incidents log from the log repository and suggesting actions based on the previous incidents' actions. The WoO semantic ontology map maintains the relation of all the ViOs which is an efficient and flexible way of maintaining collaboration, harmonization and orchestration due to the information reusability facility of semantic ontology. The WoO semantic ontology model has been described in Section 3 and the object virtualization has been discussed in Section 4. The event handler module of the EMSF is responsible for receiving fire emergency event from the ASF. On the other hand, the ASF detects fire symptoms by analyzing the temperature, gas and smoke values which are received from the sensors through the gateway. To confirm the fire incident, ASF sends a request to guard by a smart phone app to checking the suspected location physically.

We consider the guard as the person responsible for reconfirming the fire physically, but it can be any other person such as an emergency handling team member according to the service provisioning policy. After getting the confirmation from the guard application, the ASF immediately sends a notification message to all the active users.

**Figure 10.**
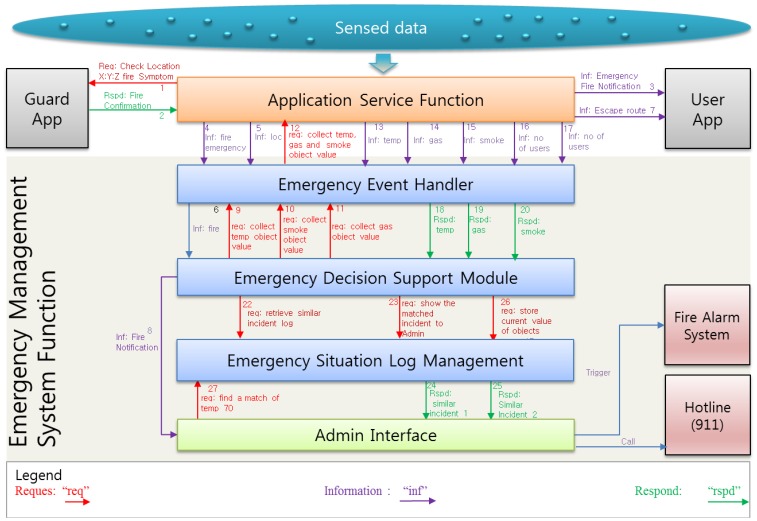
ASF rechecks the fire with the help of the guard, and notifies the EMSF and the user. The detailed message flow among all the modules during an emergency fire incident is shown in the diagram. After getting the confirmation on an admin interface, the concerned person triggers the fire alarm and calls the hotline for help.

**Figure 11.**
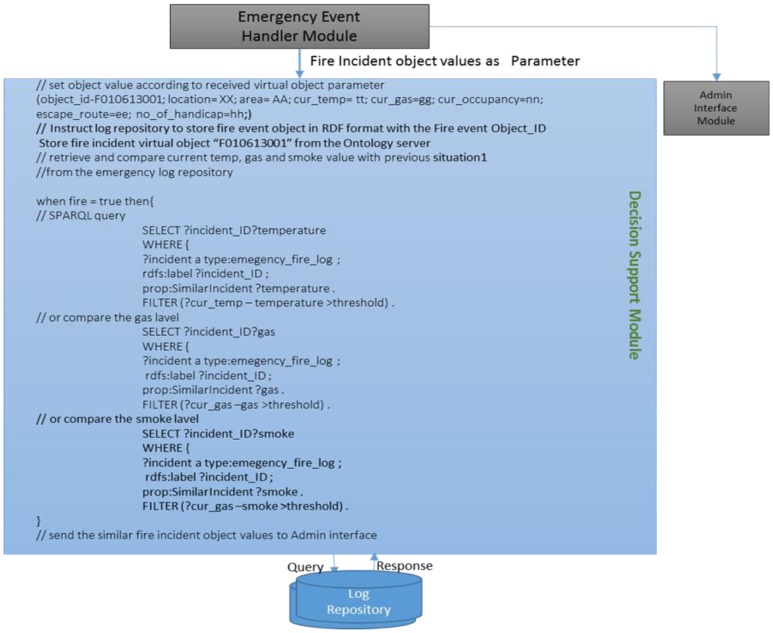
Searching similar incident in the log repository using the SPARQL query.

Henceforth, in Figure 10, the decision support module uses the current temperature, gas and smoke level values to retrieve a similar incident from the ESLM and also sends requests to show retrieved similar incident on the admin interface shown in Figure 11. It also instructs the ESLM to retrieve the previous incident log by comparing the current temperature value with the previous incidents' temperature.

Finding the similar incident in the log repository, we compare the current temperature with the previous incidents' temperature by following the below mentioned simple algorithm:

1. ASF → EMSF “Fire Emergency” // *ASF notifies EMSF*
2. If Fire event = True
3. Then
4. Current temperature=current_temperature // *collect current temperature in a variable*
5. temperature_difference = (current_temp – incident_1_temp) // *find the temperature difference*
6. If
7. temperature_difference = threshold // *threshold indicates the acceptable maximum duration*
8. Then
9. select incident_1; retrieve all the values of the incident_1 // *retrieve selected incident data*
10. else
11. go to the next incident
12. End

The ESLM module receives the temperature, gas and smoke object value as a query and returns the similar incident data as output to the admin interface. The manager is the person authorized to manage the emergency situations by accessing the admin interface. He can customize the actions according to the necessity and retrieve any required data from ESLM. ViOs are related with each other through the semantic ontology model and can provide the current object value in RDF format.

To improve on this simple principle, the size of the archive can be increased and the dimensionality and the complexity of the log repository can also be increased. To enhance the flexibility to retrieve the available situation's data, we categorize the log repository to store several incidents and actions related to different situational inputs. We believe that this classification will make the system simpler in terms of its searching and matching function.

Hence, the emergency log has three categories: user logs, fire logs, and event and actions log. The user log contains the information of users like user profile, entrance time and the current location of the user and also the total number of users at that time. The total number of users represents the population in the shopping mall at that particular time which is very important to generate an appropriate escape route.

The fire logs category encompasses the location of the fire, reason for the fire, the temperature, gas and the smoke values, the area affected by the fire and the fire occurrence time. All these data are very important to the decision maker to decide on the current actions. Moreover, the temperature, gas and smoke values are the key factors for retrieving the previous incidents from the repository.

The third category is the event and actions log. Most of the logs in this category are mainly for future analysis to improve the overall performance, such as how long it has taken to trigger the fire alarm and call 911 after the actual fire event, or how long the guard has taken to confirm the fire event, how many false fire alarms has been detected by the sensors and when the rescue team arrived, *etc*. However, these types of logs are helpful for making instant decisions as well. For example, when deciding the next action it is important to know the approximate arrival time of the rescue team.

**Figure 12.**
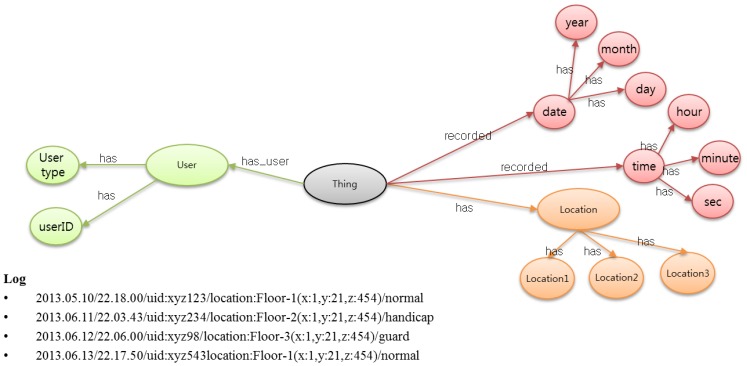
Emergency log is collected from ViOs. All the fire emergency related objects are connected with each other in the semantic ontology model.

The log categorization is required for the system to identify the actions, user and fire log independently. For example, Figure 12 shows that the user's location log is collected from the semantically related ViOs along with the relevant information. Here, the relevant information is the date, time, user-id and the current location of the user. In the same way, other incident log can also be collected from the semantic ontology model. For the fire incident log, it stores the date, time and the location describing the fire incident along with the smoke, gas and the temperature levels. These smoke, gas and the temperature values are very important to make further decisions for any other fire incident in the future. Moreover, these data can be treated as a valuable resource to evaluate the emergency management performance.

It is also important to have all the actions logged in the ESLM for analyzing the emergency fire management system's performance. Fire alarm triggering time and the hotline calling time log can be helpful for future analysis. By comparing the fire event time with the fire alarm triggering time and the hotline calling time, it is possible to understand the basic efficiency of the systems. Unexpected delays in any of those two actions indicates a fault or inefficiency in the system which can be extremely dangerous.

## 6. Prototype Implementation

**Figure 13.**
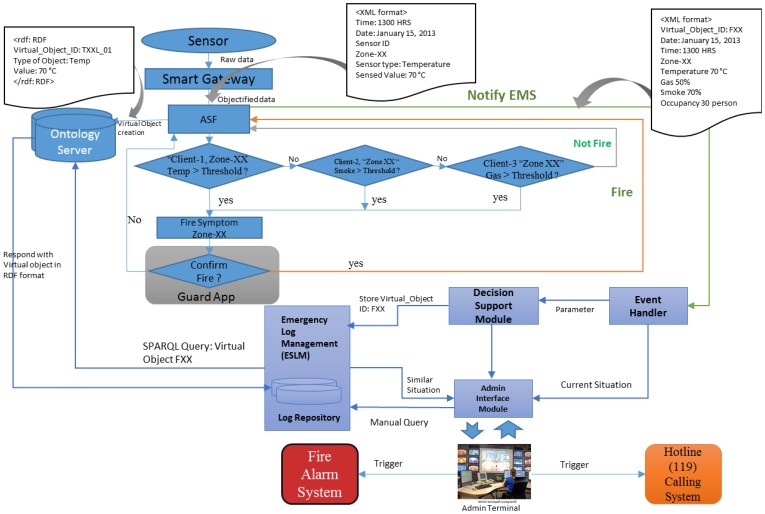
Implementation scenario in the form of flow diagram of emergency fire detection and management.

**Figure 14.**
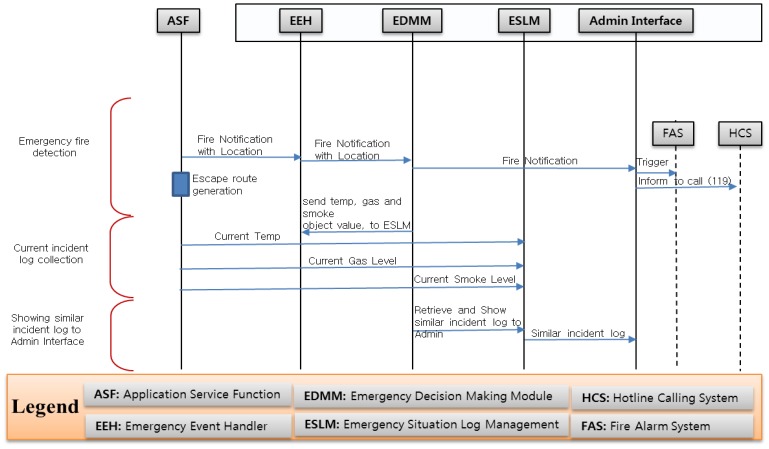
Sequence diagram for emergency fire management system.

Our implementation scenario and sequence diagram are depicted in Figures 13 and 14 respectively. Fire detection happens in the ASF. First the ASF compares the received value with a threshold to detect fire incidents and assigns a guard to check if there are any real fire indications. This is normally done automatically by the system. However, after the confirmation from the guard, the EMSF internal functions are executed, the ESLM stores current values, and the decision maker instructs the ESLM to retrieve an emergency situation log and send it to the admin interface.

Though fire detection does not happen in the EMSF, it is helpful to understand the full process after receiving the raw data from the sensors. First, the ASF compares the current temperature, smoke and gas received values with a predefined threshold to detect any fire symptoms. If there is any symptom, then the ASF assigns a guard to check for confirmation.

However, after receiving the confirmation from the guard, the EMSF's internal functions are executed, and the event handler sends the parameter of the current fire incident objects to the decision support module and the admin interface. The decision support module instructs the ESLM to retrieve the current emergency incident log from the ontology server. The ESLM only knows the virtual object id and sends requests to ontology server for collecting the current fire incidents' virtual object values.

Only authorized users can access the admin interface which has the authority to make important decisions after receiving the fire notification. In our implementation, we consider Fire Alarm and Hotline calling as a manual system and it is a responsibility of those persons who have access to the admin interface. However, it can also be integrated into the core system depending on the requirement and the capability of the fire alarm system and the hotline calling systems. Apart from the suggested incident from the EMSF, this authorized person can make customized queries to retrieve data from the log repository.

The web applications for the EMSF and ASF are developed in Java Enterprise Edition on and Eclipse platform. Several JAVA script pages are generated by the servlet to show each and every operation. We design the ontology for an emergency fire incident in Protégé. Hence, for example, sensor-node has a subclass location node, temperature node, gas node and smoke node. Defining properties such as “has temperature”, “has user”, “has location” is used for relating different classes. Data properties need to be defined in order to represent data attributes of the class. All this class has relationships with other classes in the semantic ontology model. We present a normal visualization of the semantic ontology model for fire emergency in Figure 3. However, the reader should not be confused into assuming that only direct relations are possible, rather, any of these subclasses has a relation with all other subclasses. For example, the user will have a unique ID, a user type as well as the login/registration time with the exact location. Like this, a fire incident will have all the related subclasses' information in the fire incident object.

**Figure 15.**
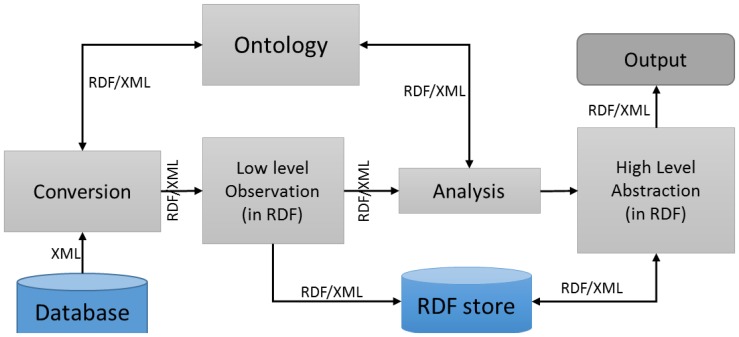
Sensors raw data needs to be abstracted in a machine usable form such as Resource Description Framework (RDF).

Hence, raw data for RDF creation using the ontology model is divided into four segments as shown in Figure 15: raw data collection from sensors through a database, conversion of raw data to RDF, analysis of raw data, and ensuring access through a graphical user interface (GUI). Environmental raw data is collected from the sensors then converted to RDF format using OWLAPI.

Sensed raw data analysis is accomplished according to the policy and rules to detect the fire symptoms, and the sensor observations are analyzed to determine the occurring fire event. In this case, an abstraction is a record of the fire event; these abstractions of events are also set in RDF. The processed high-level abstractions are used for situation awareness and decision making. The final results are demonstrated using a simple GUI that will show the fire is detected. In addition, all the RDF data is accessible through a SPARQL endpoint. The object values are represented in RDF so combining the two RDF is treated as combining the two object values; in other way, two or more objects can be combined if their RDF is combined.

**Figure 16.**
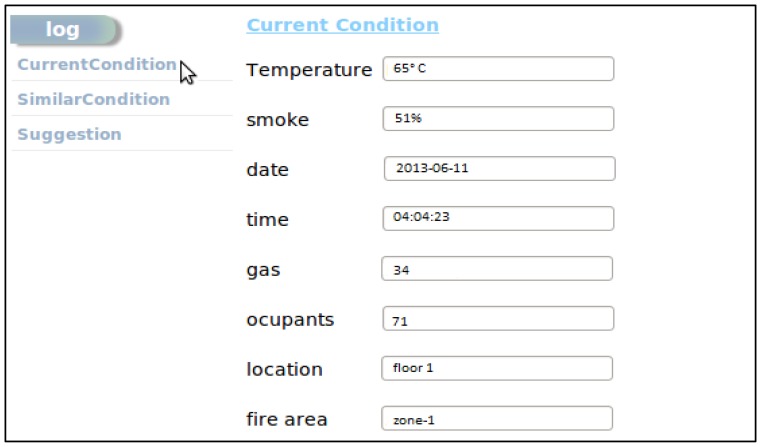
Current condition in the fire affected area.

Figure 16 shows the current conditions data in the admin interface, as we have described before that current fire conditions data is used by the system to retrieve similar types of incident from the log repository.

**Figure 17.**
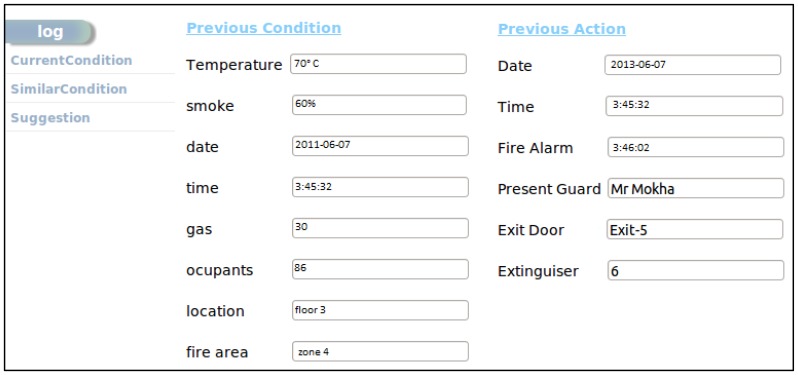
Similar conditions and actions from log repository.

Here, previous similar conditions are shown in Figure 17. In reality, exactly similar conditions are not common; rather, we are considering comparatively the closest situation.

During this work, we have identified a few issues such as service adaptation based on the context and the user profile, whether devices and services can be interoperable, whether federated query processing should be included and the virtual object should be intelligent to provide more dynamic services.

## 7. Conclusions

In this paper, we have presented a WoO-based emergency fire management system. Accordingly, we have defined real world objects in the virtual world with the intention of spanning the virtual world with the physical world as well as to satisfy the requirement of the WoO. We have presented the intellectual management and formation of ViOs. The WoO semantic ontology model maintains relationship among all the ViOs. In addition, the WoO semantic ontology model has been used to create new ViOs dynamically because of the information reusability, extensibility and interoperability advantages of the ontology model. The combination of ViOs is capable of creating new ViOs. These ViOs are capable of maintaining orchestration, harmonization and federation among them. This dynamicity facilitates smart applications that can be reused outside of the original context and domain.

We have also presented a log collection procedure from the semantically collaborated ViOs. This situation log helps fire management teams make instant decisions by providing previous fire incident logs. This log repository actually helps the concerned person/team as a decision support tool to handle fire emergencies. A symptomatic smart shopping mall scenario has been considered in order to implement and validate the proposed log based emergency management systems.

The future work plan includes the complete implementation of the WoO at a professional level that is designed but not yet implemented. Consequently, some important issues such as security, privacy and performance evaluation have not been described in this paper. We are highly motivated to address those abovementioned issues in our next work. We are also developing user applications in order to enhance the system's functionality and to ensure its completeness.
